# Recovery after cardiac arrest: the brain is the heart of the matter

**DOI:** 10.1007/s12471-018-1156-7

**Published:** 2018-09-06

**Authors:** J. Hofmeijer, M. E. W. Hemels

**Affiliations:** 1 Department of Neurology, Rijnstate Hospital, Arnhem, The Netherlands; 2 Clinical Neurophysiology, Technical Medical Center, University of Twente, Enschede, The Netherlands; 3 Department of Cardiology, Rijnstate Hospital, Arnhem, The Netherlands; 4 Department of Cardiology, Radboud University Medical Center, Nijmegen, The Netherlands

Since the 1990s, survival rates of out-of-hospital cardiac arrest have increased considerably in the Netherlands, from 16% in 2006 to 23–27% in 2016, to even 41% in patients with a shockable rhythm. In comparison, survival after cardiac arrest in the USA was 12% in 2016 [1, 2]. The exemplary increase in survival in the Netherlands is related to national programmes aimed at increasing awareness of signs of cardiac arrest, providing education on basic life support to the general population, and making available dense networks of automated external defibrillators throughout the country [1, 2]. The Dutch Heart Foundation (*Hartstichting*) has formulated the criteria for so-called ‘6-minute zones’ to save an additional 2,500 lives per year [3].

In sharp contrast with increased survival after cardiac arrest, neurological outcome has changed only marginally over the past decades. Of those surviving up to hospital admission, more than three-quarters initially remain comatose as a result of diffuse anoxic-ischaemic brain damage. Half of comatose patients die in hospital. Disturbances of motor function, cognition, mood, or other neurological impairments have been found in up to 100% of survivors [4, 5]. Cognitive impairments are strongly related to reduced quality of life [6]. Rates of mortality, anxiety, and depression appear to be higher in women than in men [2, 4].

Early recognition of disturbances of motor function, cognition or mood would allow better guidance of patients, and open avenues for targeted treatments. Accordingly, both the Dutch and the European Resuscitation Council guidelines for cardiac rehabilitation recommend screening for cognitive impairments and cognitive rehabilitation [7]. However, in patients that wake up from a coma, diagnosis and treatment are focused on cardiac function, while brain damage and neurological impairments are addressed infrequently and not systematically. Protocols to diagnose cognitive and subsequent functional impairments are scarce. There are no effective treatments to promote recovery of brain function and improve neurological outcome [8].

In this issue of the *Netherlands Heart Journal*, Boyce and co-workers assess the acceptance of the guideline recommendations amongst Dutch cardiologists and rehabilitation specialists, as well as their current implementation, by means of questionnaires [9]. The vast majority of responders acknowledged the importance of cognitive screening in cardiac arrest survivors, including the need for clear protocols. However, only a minority reported actual implementation of a cognitive screening protocol in their clinic. In addition, the authors analysed barriers to and success factors for implementation. They established the following barriers: lack of knowledge of cognitive disturbances amongst cardiologists, logistic and financial problems (that unfortunately were not further described), poor collaboration between cardiac and cognitive rehabilitation specialists, relatively small numbers of patients in some hospitals, and fear of administrative overload. Many respondents saw opportunities to implement protocols for the diagnosis and treatment of cognitive disturbances. These include more personalised treatment and a consequent decrease of drop-outs during the cardiac rehabilitation programme.

We underscore the importance of brain damage after cardiac arrest and compliment Boyce and co-workers for their efforts to draw attention to this. It is our strong opinion that, after successful programmes to increase survival rates, we now have the responsibility to build on the growing evidence of cognitive and emotional impairments to improve neurological and psychiatric diagnosis and treatment. We will have to develop and implement a rational approach for the identification of brain damage, and to test rehabilitation treatments to promote functional recovery. Until further evidence becomes available, screening for cognitive impairments may be performed using the Montreal Cognitive Assessment, which takes a trained nurse just 10 min [9]. In the presence of relevant cognitive disturbances, cardiac rehabilitation may include psycho-education and strategy training. In this way, even a little effort may result in significant improvement of patient-oriented rehabilitation of survivors after cardiac arrest.
